# Mediterranean monk seal (*Monachus monachus*) and leopard seal (*Hydrurga leptonyx*) de novo genomes to study the demographic history and genetic diversity of southern seals

**DOI:** 10.1186/s12915-025-02207-w

**Published:** 2025-04-16

**Authors:** Marcel Nebenführ, Alexander Ben Hamadou, Alex Aguilar, Asunción Borrell, Georgios A. Gkafas, Axel Janke

**Affiliations:** 1 Institute for Ecology, Evolution and Diversity, Goethe University, Max-Von-Laue-Strasse 9, Frankfurt Am Main, Germany; 2 LOEWE-Centre for Translational Biodiversity Genomics (TBG), Senckenberg Nature Research Society, Georg-Voigt-Straße 14 - 16, Frankfurt Am Main, Germany; 3 Senckenberg Biodiversity and Climate Research Centre (BiK-F), Georg-Voigt-Straße 14 - 16, Frankfurt Am Main, Germany; 4 Department of Evolutionary Biology, Ecology and Environmental Sciences, and Institute of Biodiversity Research (IRBio), University of Barcelona, Diagonal 643, Barcelona, 08009 Spain; 5 Department of Ichthyology and Aquatic Environment, School of Agricultural Sciences, University of Thessaly, Volos, Greece

**Keywords:** Leopard seal, Mediterranean monk seal, Pinnipedia, Seals, Genome assembly, Genome annotation, Demography, HiFi sequencing

## Abstract

**Background:**

The Monachinae, or southern seals, are one of two subfamilies within the Phocidae and are home to iconic pinnipeds such as the leopard seal, a fierce Antarctic top predator, and the Mediterranean monk seal, one of the world’s most endangered mammals. These two species are difficult to study and sample, due to their hidden lives in extreme environments or, in case of the monk seal, their critically reduced population sizes; consequently, genetic data from these two species is scarce. However, cost developments and advances in genome sequencing have made it possible to generate continuous genome assemblies from DNA of even stranded individuals, allowing to assemble the first reference genomes of such rarely observed species.

**Results:**

In this study, we have sequenced the genomes of the leopard seal and the Mediterranean monk seal using PacBio’s CCS technology to assemble the very first genomes for these species. Four additional Mediterranean monk seal individuals were sequenced using Illumina short-read technology. These data allowed analysis of their demography and genomic diversity based on whole-genome data, confirming low genetic variability and small numbers of individuals for the Mauritanian population of the Mediterranean monk seal. In contrast, the relatively abundant leopard seal shows a high degree of heterozygosity, comparable in the range of other common carnivores.

**Conclusions:**

The first genome assemblies for these seals will lay the groundwork for population-level and other studies to better understand their evolutionary history and biology and to aid conservation efforts.

**Supplementary Information:**

The online version contains supplementary material available at 10.1186/s12915-025-02207-w.

## Background

The Phocidae, or true seals, are divided into two subfamilies reflecting their current biogeography: the southern seals (Monachinae) and the northern seals (Phocinae). The Monachinae consist of eight recent species that have diverged from their sister lineage approximately 12 Ma ago and are found in numerous environments [1–4]. Monk seals are the only extant phocid seals that inhabit warm, tropical environments, while all remaining Monachinae seals, including the leopard seal, an Antarctic top predator, are found in the cooler waters of the Southern Hemisphere [5–7].

Even though the Monachinae contain captivating species of major scientific interest, they are vastly understudied at the genetic level. Molecular analyses of their phylogeny and genetic diversity have been limited to mitochondrial genomes [1, 8] or a few nuclear loci [5]. Genome assemblies are still missing for iconic species like the leopard seal or the Mediterranean monk seal — two species that have been the subject of research for decades, due to their importance to ecosystems as top predators or their conservation status.

The leopard seal (*Hydrurga leptonyx*) is an abundant species, distributed along the Antarctic ice sheet, with sightings reported from as far north as Australia, New Zealand, and South Africa [9]. It is the largest of the four Antarctic seal species, reaching lengths of over 3 m and weighing up to 600 kg. As a top predator of Antarctica, it influences its marine ecosystem through top-down control [9, 10]. Although leopard seals are known for preying on penguins, their diverse and seasonally variable diet includes krill, fish, and mollusks, as well as juveniles of other seal species [9, 11, 12]. Their predation is known to affect the population sizes of Antarctic fur seals (*Arctocephalus gazella*) and has been reported to cause population collapses at Cape Shirreff on the northern Antarctic Peninsula [13, 14].

While the leopard seal is an abundant species, the Mediterranean monk seal (*Monachus monachus*) is considered as one of the world’s most endangered mammals. It is the only extant species of the monotypic genus *Monachus* and reaches lengths of about 3 m and weighs up to 400 kg [6, 15, 16]. In its habitat, this non-migratory seal lives in a solitary social organization, mother–pup relationship, or in smaller groups, and breeds on islands, remote mainland beaches, and sea caves [16, 17].

The degradation of these habitats is one of many reasons for the severe decline of the Mediterranean monk seal populations. In addition to habitat loss, the species’ past is shaped by a long history of exploitation that has depleted the populations or driven them to local extinction.

Hunting of the Mediterranean monk seal for its fur, oil, and pelts dates back to the Stone Age and reached substantial levels during the era of the Roman Empire, which already reported a notable decline in the population [18, 19]. In the twentieth century, deliberate killing by fishermen, habitat deterioration or loss (e.g., through pollution or tourism), and a mass die-off at the Cabo Blanco monk seal colony became the main threats to the species, leading to a dangerous population decline of the Mediterranean monk seal [19, 20]. As a result, the distribution of this species, which once occupied a large geographical range in the Black Sea, Mediterranean Sea, and Atlantic coasts, is now restricted to small areas in the northeastern Atlantic and the eastern Mediterranean Sea. Its total population is now estimated to be about 500 individuals [20, 21].

The results of the severe and steady population decline and the extended bottleneck are already evident at the genetic level. A microsatellite study of 52 Mediterranean monk seals found that genetic variation was among the lowest reported for any mammal and shows no population structure between different nursing caves [22]. A mtDNA-based study on the populations in the eastern Mediterranean regions corroborates that this species is one of the most genetically depauperate mammals [23]. Therefore, estimating genetic parameters relevant for conservation on an extended, genome-wide dataset is essential to assess the extent of genetic depletion at nuclear loci and to aid conservation efforts for this unique seal species.

In this study, we sequenced and de novo assembled the first genomes of the leopard seal and the Mediterranean monk seal using PacBio CCS long-read technology. Four additional individuals of the Mediterranean monk seal were sequenced using Illumina short-read technology. The generation of these data allowed assessing the phylogeny and genomic diversity across the southern seals based on genome-wide data and to estimate their demographic history and divergence times.

## Results

### Genome assembly

**Fig. 1:**
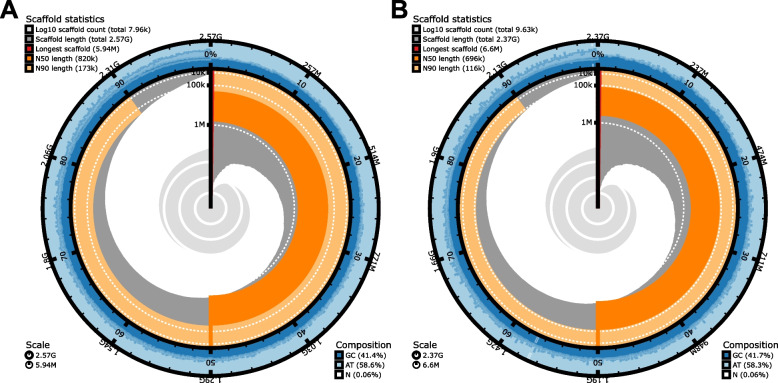
Assembly features and essential quality measures of the de novo genomes of the leopard seal and the Mediterranean monk seal summarized in a snail plot. The innermost circle shows scaffold statistics, and the colors from red to orange indicate the longest scaffold, N50, and N90. The GC composition is shown in the outer blue circle

**Fig. 2:**
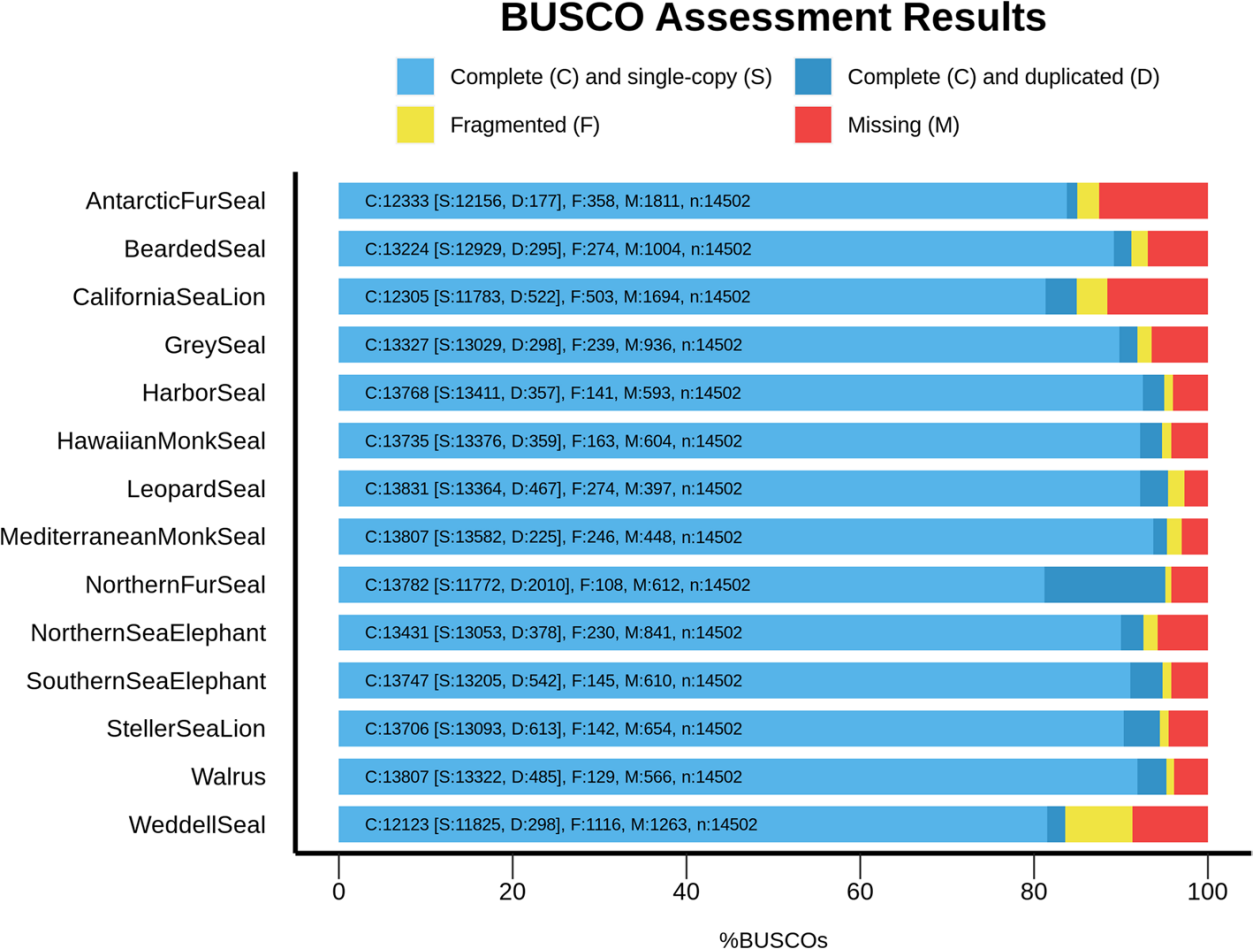
Comparison of BUSCO completeness statistics based on the Carnivora database between our final de novo leopard seal and Mediterranean monk seal genomes and selected available seal assemblies. The x-axis shows the BUSCO completeness in percent. Complete single-copy genes are shaded light blue; complete duplicated sequences are shaded blue. Fragmented genes are shaded yellow, and missing sequences are shaded red. The numbers of complete single-copy (**S**), complete duplicated (**D**), fragmented (**F**), and missing genes (**M**) for the respective genome are shown in each column. The total number of genes in the BUSCO Carnivora library is denoted as n

The three PacBio sequencing runs of the leopard seal DNA yielded a total of ~ 64 Gbp of long-read sequence data resembling a 26-fold coverage, with an average subread N50 of ~ 13 kbp. The genome of the leopard seal was assembled to a total length of 2.57 Gbp with 5120 contigs and an N50 of 868 kbp (Fig. 1A). Gene completeness analysis of the genome based on BUSCO’s Carnivora dataset identified 13,489 complete single-copy orthologous sequences corresponding to 93.01% completeness and 447 (3.08%) missing genes (Fig. 2).

The two sequencing runs of the Mediterranean monk seal DNA yielded a total of ~ 49 Gbp of long-read sequence data resembling a 21-fold coverage, with an average subread N50 of ~ 6.2 kbp. The genome was assembled to a total length of 2.31 Gbp with 8106 contigs and an N50 of 715 kbp (Fig. 1B). Gene completeness analysis of the genome based on the BUSCO Carnivora dataset identified 13,610 complete single-copy orthologous sequences corresponding to 93.85% completeness and 461 (3.18%) missing genes (Fig. 2).

Repeat annotation of the leopard seal genome identified 37.1% of repeats, of which the majority consisted of LINEs (24.9%), SINEs (2.6%), and DNA transposons (2.3%). In addition, 1.4% of unclassified repeats were identified (Additional file 1: Table 1).

Furthermore, repeat annotation of the Mediterranean monk seal genome identified 40.1% of repeats, of which the majority consisted of unclassified repeats (27.4%), LINEs (7.7%), and SINEs (1%). In addition, 27.4% of the repeats were unclassified (Additional file 2: Table 2).

Gene annotation of the leopard seal genome identified 27,054 genes and 57,693 transcripts. Annotation completeness analysis using BUSCO in protein mode identified 12,410 complete orthologous sequences corresponding to 85.6% completeness and 1639 missing genes (11.3%) (Additional file 3: Table S3).

Gene annotation of the Mediterranean monk seal genome identified 25,765 genes and 56,106 transcripts. Annotation completeness analysis using BUSCO in protein mode identified 12,497 complete orthologous sequences corresponding to 86.1% completeness and 1580 missing genes (11%) (Additional file 3: Table S3).

### Genetic diversity and demographic history

**Fig. 3:**
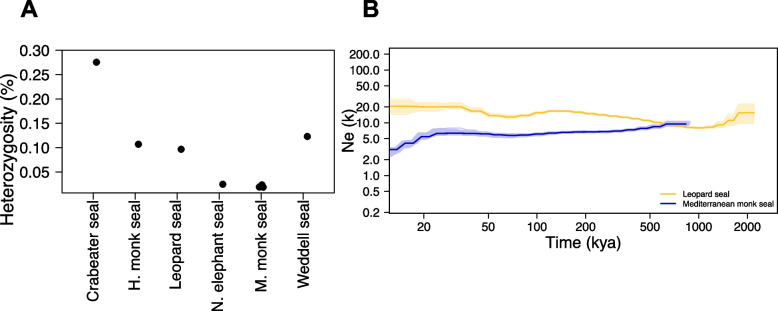
**A** Genome-wide heterozygosity. **B** Estimated historical population size (*N*_e_) of the Mediterranean monk seal and the leopard seal using PSMC analysis. The x-axis represents time, and the y-axis represents the effective population size (*N*_e_). Plots were scaled using a mutation rate (μ) of 0.7 × 10^ − 8 substitutions per nucleotide per generation and a generation time (g) of 11 years for the leopard seal and 10 years for the Mediterranean monk seal

The genome-wide heterozygosity varied considerably among all species (Fig. 3A). Estimates were lowest for the Mediterranean monk seal with a mean of 0.014% (0.14 heterozygous sites/kbp) and the northern elephant seal with 0.017% (0.17 heterozygous sites/kbp). The highest values were found for the crabeater seal (*Lobodon carcinophaga*) with 0.27% (2.7 heterozygous sites/kbp), followed by the Weddell seal (*Leptonychotes weddellii*) with 0.116 (1.16 heterozygous sites/kbp), the Hawaiian monk seal (*Neomonachus schauinslandi*) with 0.106% (1.06 heterozygous sites/kbp), and the leopard seal with 0.091% (0.91 heterozygous sites/kbp).

The history of the effective population size (*N*_e_) over the last one million years (Ma) was modeled from the distribution of heterozygous sites across the genome using a pairwise sequential Markovian coalescent (PSMC) analysis. Ancestral effective population sizes for the Mediterranean monk seals were higher around 1 Ma and kept on decreasing until present times (Fig. 3B). From approximately 50 thousand years (ka) ago, the decline in their *N*_e_ became steeper. For the leopard seal, the analysis shows an increase in *N*_e_ from approximately 2 Ma until 200 ka, followed by a decline until 100 ka. Afterwards, the *N*_e_ stabilized and started to increase until ~ 20 kyr ago.

### ML phylogeny and divergence time estimation

A supermatrix phylogeny based on an alignment of 8588 protein-coding genes was constructed using available seal genomes plus the two newly sequenced species. The final dataset included all species of Monachinae except the Ross seal, two northern seal species, and two species of the eared seals, and the walrus. The tree was rooted at the branch containing the eared seals and the walrus. The resulting phylogeny shows monophyletic clades for the monk seals, elephant seals, and the Lobodontini group, represented by the leopard seal and Weddell seal, with the monk seals being the outgroup to the remaining southern seals. Further, all representatives of the northern seals form a sister clade to the southern seals (Additional file 4: Fig. S4).

**Fig. 4:**
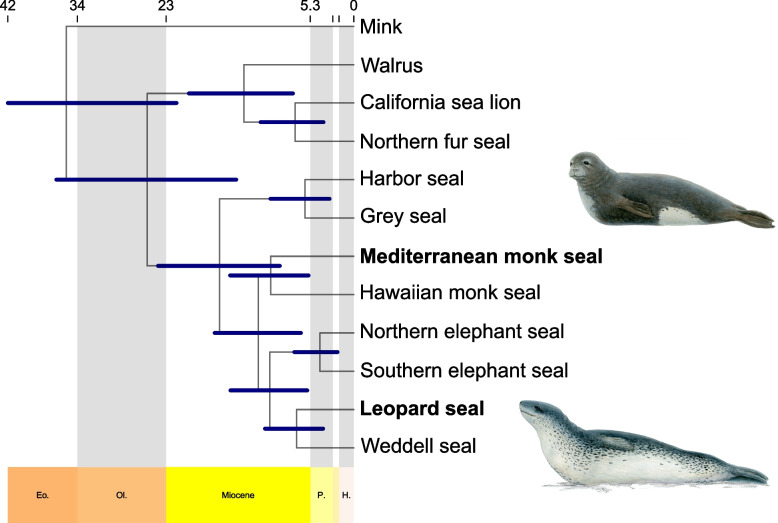
Pinnipedian divergence time tree including the newly sequenced southern seals, estimated from 2,635,559 amino acid sites (8647 orthologs). The scale on top of the plot shows absolute ages in million years, and dark blue bars indicate plot node uncertainty by highlighting the upper and lower highest posterior density of each age. Gray shading is used to delimit geologic periods

Divergence time estimates based on four calibration points were used for dating the phylogeny to generate the first divergence time estimates for Pinnipedia based on whole-genome data (Fig. 4). The analysis shows that the southern seals diverged from the northern seals in the early Miocene, approximately 21.4 (19.4–40.8) Ma ago. The monk seals, with the Hawaiian and the Mediterranean monk seal as the only extant species split from the remaining southern seals in the Miocene approximately 12.7 (10.7–27.8) Ma ago. Within the monk seals, the split between the Mediterranean monk seal and the Hawaiian monk seal took place in the late Miocene, at approximately 8.2 (3.6–13.9) Ma ago. In addition, the split between the northern and southern elephant seals occurred in the late Miocene to early Pleistocene epoch at approximately 2.9 (1.2–5.6) Ma ago.

## Discussion

The de novo assemblies of the Mediterranean monk seal and the leopard seal provide the first genomes for these species which allow for analyzing their phylogeny, demographic history, and genetic variability on genome-wide data.

Due to the difficulty of obtaining high-quality DNA samples from these species, which are either rare or elusive, chromosome-level resolution could not be achieved for the genomes. Yet, PacBio HiFi sequencing technology still allowed for continuous and complete genomes being in the range of other available seal genomes. Gene completeness analysis for the leopard seal and Mediterranean monk seal genomes show the number for complete orthologous sequences is among the highest of available seal genomes.

### Genetic diversity and population size history

Sliding window genetic diversity calculations indicate that the genome-wide heterozygosity of all species analyzed is within the range of previous heterozygosity calculations for phocid seals based on genome-wide data [24]. Moreover, the results showing the highest heterozygosity for the crabeater seal and similarly low values for the leopard seal and Weddell seal support previous genetic diversity calculations based on mitochondrial control region sequences among Lobodontini seals [25]. The exceptionally low values for the Mediterranean monk seals aid previous estimates based on multiple microsatellite loci that found alarmingly low genetic variation in this species, being among the lowest in pinnipeds [21, 26, 27]. Moreover, even in comparison to other marine or carnivorous mammalian species, the heterozygosity of the Mediterranean monk seal only falls behind the vaquita [28] and the San Nicolas Island fox [29, 30].

PSMC analysis shows no evidence of bottlenecks in the demographic past of the leopard seal for the past two million years. In contrast, its stable *N*_e_ began to increase from about 80 ka ago in the Late Pleistocene epoch to ~ 20 kyr ago. This trend was already observed by [25] and supports their discovery of a population expansion of the leopard seal in the late Pleistocene epoch based on a Bayesian skyline plot. Further, they estimated its present *N*_e_ using a Bayesian most probable estimate (MPE) to be at approximately 16.9–33.1 thousand extant individuals. These findings are in the range of both PSMC results and IUCN data, assuming a total number of 35,500 (10,900–102,600) extant leopard seals [9, 25], highlighting the wide distribution and high abundance of this species.

PSMC analysis for the Mediterranean monk seal population shows a stable *N*_e_ from 600 ka ago to ~ 20 ka ago. Thereafter, the plot shows a steady population decline until the present which is consistent with other studies showing the same pattern for this species. Even though PSMC results become unreliable for the interval from ~ 20 ka ago until present times [31], together with the low genetic diversity estimates, the results emphasize the threatened situation of the decimated Mediterranean monk seal populations and underline the importance of strict conservation actions proposed by [23] to protect this endangered species from anthropogenic threats.

### ML phylogeny and divergence time estimation

Phylogenomic inference of the Monachinae group based on 2,635,559 amino acid sites from 8647 orthologous protein-coding sequences shows that the monk seals and elephant seals form separate clades from the remaining Antarctic seal species (Lobodontini), with the monk seals being the outgroup to all other southern seals. These results are all consistent with previous phylogenetic studies based on mitochondrial and nuclear loci [1, 32]. All branches were unambiguously supported by 1000 bootstrap replicates. Together with the congruent phylogenies of the different datasets, this indicates low conflict in the data with no signals for a speciation under gene flow or the presence of considerable amounts of incomplete lineage sorting. Further, no mitochondrial capture has occurred.

Bayesian divergence time dating based on the same sequence alignment proposes the first pinniped divergence times based on a whole-genome dataset, whereas to date they have only been assessed using datasets consisting of mtDNA data [1] or a combined dataset including mtDNA and a few nDNA loci [33], respectively.

With 25.3 Ma, the estimated best divergence time for the basal Pinnipedia split based on genome-wide data is in the range of calculations based on the combined dataset consisting of mtDNA and 15 nDNA loci that found this split to have occurred approximately 22.6 Ma ago [33]. In contrast to that, the mtDNA dataset found the best divergence time for the Otaroidea and Phocidae split to have occurred further in the past, at approximately 33 Ma ago [1].

Genome-wide divergence times show that the split between the northern seals (Phocinae) and southern seals (Monachinae) happened at approximately 16.5 Ma ago, which again is closer to the combined mtDNA and nDNA dataset that estimated this split to have happened at 14.7 Ma ago [33], contrary to the estimated split time based on only mtDNA of approximately 22 Ma ago [1]. For the split between the two extant *Monachus* species, genome-wide divergence time estimates show a split time of about 10.2 Ma which lies between the mtDNA best split time at approximately 13.4 Ma and the estimate of the combined dataset that is at approximately 6.4 Ma.

## Conclusion

With this study, we report the first genome assemblies of two seal species which are of scientific interest. The leopard seal is a widely distributed apex predator that strongly influences its ecosystem through top-down control. The Mediterranean monk seal is considered as the world’s most endangered pinniped. Reference genomes are important resources as they serve as cornerstones for follow-up studies based on large-scale population data to better understand the seal’s population status and thus aid conservation efforts.

## Methods

### Sampling, DNA extraction, and genome sequencing

Tissue samples of heart, liver, kidney, muscle, intestine, skin, and blubber from five stranded individuals of the Mediterranean monk seal (Additional file 5: Table S5) were collected by AA and AB during the mass die-off that decimated the Western Sahara-Moroccan population in spring 1997 [34]. The collection and export of the samples to Spain was conducted with the permission and collaboration of the Mauritanian Parc National du Banc d’Arguin, the local wildlife authority in the region.

The sampling was part of the Emergency Rescue Action launched by the European Union, the coordination of which was the Ministry of the Environment of Spain, and the beneficiary was the Government of the Canary Islands in partnership with the Banc National du Banc d’Arguin. The Emergency Rescue Action was supervised by the IUCN Pinniped Working Group. At that time, 30 years ago, Mediterranean monk seals were dying daily by unknown reasons, so the samples were collected and transported in urgency and distributed to various institutions in Europe for further analyses. Since Mauritania was not then signatory of CITES, all institutions involved agreed to proceed swiftly without consideration to the CITES procedures. The monk seal samples in the underlying study were used before in at least eleven successful research projects involving European and US researchers that have been published in well-reputed scientific journals (Additional file 6: Table S6).

The collection area was the Las Cuevecillas coast, where the main hauling-out caves are located [35]. Tissues were stored at − 80 °C and preserved in ethanol for shipment. High molecular weight DNA (hmwDNA) was extracted from the samples using the QIAGEN DNeasy Blood and Tissue Kit (QIAGEN N.V., Hilden, Germany). DNA quality and quantity were assessed using the Genomic DNA ScreenTape on the Agilent 2200 TapeStation system (Agilent Technologies) and the Qubit fluorometer. The geographic origin of the leopard seal hmwDNA sample has unfortunately not been recorded and is the same sample that was used in [1, 36].

Based on the DNA quality, sample “M- 2” was selected to generate the reference genome. The degree of fragmentation did not allow direct long-read sequencing. Therefore, a pulsed-field size selection was performed on the DNA using the BluePippin (Sage Science, Beverly, MA, USA) to eliminate DNA fragments ≤ 4 kb prior to library preparation.

Libraries were prepared for long-read sequencing using the SMRTbell Express Template Prep Kit 3.0 following the protocol for HiFi libraries from low DNA input. The libraries were sent to a local NGS sequencing provider (Bioscientia Labor Ingelheim, Ingelheim, Germany) and sequenced on a PacBio Revio system (Pacific Biosciences, Menlo Park, CA, USA).

DNA isolates from the additional monk seals and the leopard seal were sent to BGI Genomics Europe (Warsaw, Poland) to generate 150 PE Illumina re-sequencing libraries for reference-based assembly.

### Genome assembly

The Mediterranean monk seal and the leopard seal de novo genomes were assembled using Hifiasm v0.18.8-r525 [37]. Afterwards, the raw assemblies were polished using Inspector v1.0.1 [38] and scaffolded by running the full *tigmint-ntLink-arks* pipeline of LongStitch v1.0.4 [39]. Subsequently, scaffold gaps were removed using TGS-GapCloser v1.2.1 [40] without further polishing of sequencing reads. The genomes were then scanned for contamination using the run_gx.py option of FCS-GX v0.4.0 [41]. Contaminated sequences were then trimmed, excluded, or split using the *gx clean-genome* option. Finally, duplicated contigs were removed using purge_dups v1.2.5 [42]. Assembly continuity statistics of the final genomes were then calculated using QUAST v5.0.2 [43]. In addition, a gene set completeness analysis of our genomes and other seal genomes used in this study was performed using BUSCO v5.4.22 [44] with the provided database for Carnivora orthologous genes.

### Genome annotation

To annotate repetitive sequences in the genome, a custom de novo repeat library was created using RepeatModeler v2.0.1 [45] and combined with the mammals repeat database from RepBase (https://www.girinst.org/repbase/). Repeats were then annotated using RepeatMasker v4.1.2-p1 [46].

The genome annotation was carried out with GeMoMa v1.9 [47], using other available and annotated seal genomes as references (Additional file 7: Table S7).

### Reference-based assemblies and genotype calling

Sequencing data for additional seal species were collected from the NCBI database (https://www.ncbi.nlm.nih.gov/) (Additional file 6: Table S6). Reads were trimmed and filtered using Trimmomatic v0.32 [48] with the following settings: *ILLUMINACLIP:TruSeq3-PE- 2.fa:2:30:10 SLIDINGWINDOW:4:20 MINLEN:40 TOPHRED33*. Clean reads were then mapped to the leopard seal reference genome using BWA v0.7.17-r1188 r1188 [49] and Samtools v1.15 [50]. Duplicate reads were removed using the Picard MarkDuplicates software v3.1.1 (http://broadinstitute.github.io/picard/). All files were filtered for mapping quality and alignment score using Samtools view with the following settings: *-bhq 20 -f 0* × *2 -F4 -e'[AS]* > = *100'*. Finally, repetitive regions were removed from all files with the help of Bedtools intersect v2.30.0 [51]. The quality of the final files was assessed using Qualimap v2.2.2-dev [52] (Additional file 8: Table S8).

For downstream SNP-based analyses, genomic variants were called for all five Mediterranean monk seals, the leopard seal, the northern and southern elephant seal, the crabeater seal, the walrus, and the Weddell seal. Genotype likelihoods and calls were generated using the bcftools mpileup and call pipeline of BCFtools v1.12 [50], following the BAM2 VCF_run_mpileup_parallel_HIGHWAY script of the Fastq2VCF pipeline (https://github.com/mennodejong1986/Fastq2VCF) with default settings. During genotype calling (bcftools call), the “group-samples” option was used to assign each individual to its unique group, to disable the option of influencing genotype calls based on information from other samples. Sites with read depth below three were masked using the bcftools filter pipeline. For all 11 individuals combined, sites with a minimum read depth of 66 (resembling 6 × the number of samples) and a maximum depth of 225 (resembling 1.5 times the mean depth of the samples) were retained.

### Genome-wide heterozygosity and demographic history

For the population genetic analysis, the assembly of the Mediterranean monk seal was scaffolded using the available genome of the Hawaiian monk seal as reference to detect and remove sex-linked chromosomes (Additional file 6: Table S6). Trimmed sequencing reads of all species were mapped against the resulting genome without sex chromosomes the same way as described above.

The custom-built Darwindow tool [53], based on the Tabix software [54], was used to count the number of retained homozygous and heterozygous sites per sample on a sliding-window basis, using non-overlapping windows with a fixed size of 20 kb. Using Darwindow, genomic regions were extracted and converted to heterozygosity (He) estimates.

The historical effective population size (*N*_e_) of the Mediterranean monk seals and the leopard seals was estimated using Pairwise Sequentially Markovian Coalescent (PSMC) v0.6.5-r67 [31]. To do this, consensus genome sequences were generated from the reference-based assemblies using BCFtools v1.14 mpileup and filtered to remove sites with mapping quality < 30 and read depth < 10 or above twice their mean depth. PSMC analysis was run with 25 iterations and 100 rounds of bootstrapping using the default atomic interval set for humans. To scale the analysis, a generation time of 11 years for the leopard seal and 10 years for the Mediterranean monk seal was used, according to IUCN (https://www.iucnredlist.org/), and a mutation rate of 0.7 * 10^ − 8 substitutions per nucleotide and generation was chosen [55].

### ML phylogeny and divergence time estimation

The GEMOMA-to-Phylogeny pipeline (https://github.com/mag-wolf/GEMOMA-to-Phylogeny) was followed to generate a phylogeny based on single-copy orthologous sequences (SCOS). Therefore, GeMoMa was run to perform homology-based annotations of all assemblies using available seal proteomes for the species in our analysis. SCOS were then searched using OrthoFinder v2.5.2 [56] using default options and the MSA method for gene-tree inference. Alignments were then constructed with MAFFT v7.505 [57] with thresholds allowing for 5–40% variable sites. Subsequently, FASconCAT v1.04 [58] was used to concatenate all alignments to a single matrix that was afterwards trimmed with ClipKIT v1.3.0 [59] for informative and conserved sites, allowing for additional gap trimming using the “*-m kpic-smart-gap" *flag. The resulting matrix was then used to calculate branch lengths using IQTree.

The resulting tree and sequence files were used as input data for MCMCtree v4.9e [60] to investigate ancient divergence times of the included seal species. Divergence times were based on four calibration times (Additional file 9: Table S9). The analysis was run twice following the workflow for assessing divergence times based on amino acid sequences. In each run, the first 60,000 iterations were discarded as burnin, afterwards every 5 iterations were sampled until 600,000 samples were gathered. Both runs were then checked for convergence.

## Supplementary Information

Additional file 1: Table S1 Repeat content of the leopard seal.

Additional file 2: Table S2 Repeat content of the Mediterranean monk seal.

Additional file 3: Table S3 Annotation completeness of the newly sequenced genomes.

Additional file 4: Fig. S4 Maximum likelihood phylogeny.

Additional file 5: Table S5 Mediterranean monk seal sample overview.

Additional file 6: Table S6 Publication record monk seal samples.

Additional file 7: Table S7 Seal genomes and genome data taken from databases.

Additional file 8: Table S8 Mapping quality of reference-based assemblies.

Additional file 9: Table S9 Calibration points used for dating the phylogeny.

## Data Availability

We declare our intention to deposit raw sequencing reads and the genome assemblies at the National Center for Biotechnology Information (NCBI). All other data needed to evaluate the conclusions of the paper are present in the paper and/or the Supplementary Materials. Additional data related to this study may be requested from the authors.
