# Supplementary material for: Mediterranean monk seal (Monachus monachus) and leopard seal (Hydrurga leptonyx) de novo genomes to study the demographic history and genetic diversity of southern seals
Source: BMC Biol. 2025 Apr 16;23:102. doi: 10.1186/s12915-025-02207-w (PMC12004778; doi:10.1186/s12915-025-02207-w)
Supplement: Supplementary file 1 — Additional file 1: Table S1 Repeat content of the leopard seal. [file 12915_2025_2207_MOESM1_ESM.docx]

**Supplementary Table 1 Repeat content of the leopard seal genome assembly.** Class, class of the repetitive regions. Count, number of occuences of the repetitive region. bpMasked, number of base pairs masked; %masked, percentage of base pairs masked. LINE, Long Interspersed Nuclear Elements (include retroposons); LTR, Long Terminal Repeat elements (including retroposons); SINE, Short Interspersed Nuclear Elements; RC, Rolling Circle.

| **Class** | **Count** | **bpMasked** | **%masked** |
| --- | --- | --- | --- |
| SINEs | 500,642 | 67,144,614 | 2.61 |
| LINEs | 1,974,781 | 639,592,433 | 24.88 |
| LTR | 425,554 | 108,851,357 | 4.23 |
| DNA transposons | 352,610 | 58,022,310 | 2.26 |
| Rolling-circles | - | - | - |
| Unclassified | 208,976 | 36,740,814 | 1.43 |
| small RNA | 66,507 | 7,916,831 | 0.31 |
| Satellites | 20,150 | 2,123,800 | 0.08 |
| simple repeats | 610,062 | 26,952,502 | 1.05 |
| Low complexity | 100,350 | 5,268,346 | 0.20 |
