# Supplementary material for: Mediterranean monk seal (Monachus monachus) and leopard seal (Hydrurga leptonyx) de novo genomes to study the demographic history and genetic diversity of southern seals
Source: BMC Biol. 2025 Apr 16;23:102. doi: 10.1186/s12915-025-02207-w (PMC12004778; doi:10.1186/s12915-025-02207-w)
Supplement: Supplementary file 2 — Additional file 2: Table S2 Repeat content of the Mediterranean monk seal. [file 12915_2025_2207_MOESM2_ESM.docx]

**Supplementary Table 2 Repeat content of the Mediterranean monk seal genome assembly.** Class, class of the repetitive regions. Count, number of occuences of the repetitive region. bpMasked, number of base pairs masked; %masked, percentage of base pairs masked. LINE, Long Interspersed Nuclear Elements (include retroposons); LTR, Long Terminal Repeat elements (including retroposons); SINE, Short Interspersed Nuclear Elements; RC, Rolling Circle.

| **Class** | **Count** | **bpMasked** | **%masked** |
| --- | --- | --- | --- |
| SINEs | 230,811 | 24,743,048 | 1.04 |
| LINEs | 1,151,744 | 183,272,994 | 7.73 |
| LTR | 179,181 | 41,152,249 | 1.74 |
| DNA transposons | 114,895 | 16,747,038 | 0.71 |
| Rolling-circles | 5,180 | 2,907,594 | 0.12 |
| Unclassified | 2,069,949 | 648,822,028 | 27.4 |
| small RNA | 31,580 | 2,411,937 | 0.10 |
| Satellites | 14 | 378 | 0.01 |
| simple repeats | 559,565 | 25,780,779 | 1.09 |
| Low complexity | 87,220 | 4,572,633 | 0.19 |
