# Supplementary material for: Mediterranean monk seal (Monachus monachus) and leopard seal (Hydrurga leptonyx) de novo genomes to study the demographic history and genetic diversity of southern seals
Source: BMC Biol. 2025 Apr 16;23:102. doi: 10.1186/s12915-025-02207-w (PMC12004778; doi:10.1186/s12915-025-02207-w)
Supplement: Supplementary file 3 — Additional file 3: Table S3 Annotation completeness of the newly sequenced genomes. [file 12915_2025_2207_MOESM3_ESM.docx]

**Supplementary Table 3** BUSCO protein mode genome completeness results for the annotation of the Mediterranean monk seal and leopard seal genomes using the provided database for carnivores (carnivora_odb10).

| **BUSCO groups found** | | **Mediterranean monk seal** | | **Leopard seal** |
| --- | --- | --- | --- | --- |
| Complete  (S=single-copy,  D=duplicated) | 12,497 (86.1%)  S=5,966 (41.1%), D=6,531 (45%) | | 12,410 (85.6%)  S=5,768 (39.8%), D=6,642 (45.8%) | |
| Fragmented | 425 (2.9%) | | 453 (3.1%) | |
| Missing | 1,580 (11%) | | 1,639 (11.3%) | |
| Total | 14,502 | | 14,502 | |
