# Supplementary figures and images for: Mediterranean monk seal (Monachus monachus) and leopard seal (Hydrurga leptonyx) de novo genomes to study the demographic history and genetic diversity of southern seals
Source: BMC Biol. 2025 Apr 16;23:102. doi: 10.1186/s12915-025-02207-w (PMC12004778; doi:10.1186/s12915-025-02207-w)

Tree scale: 0.01

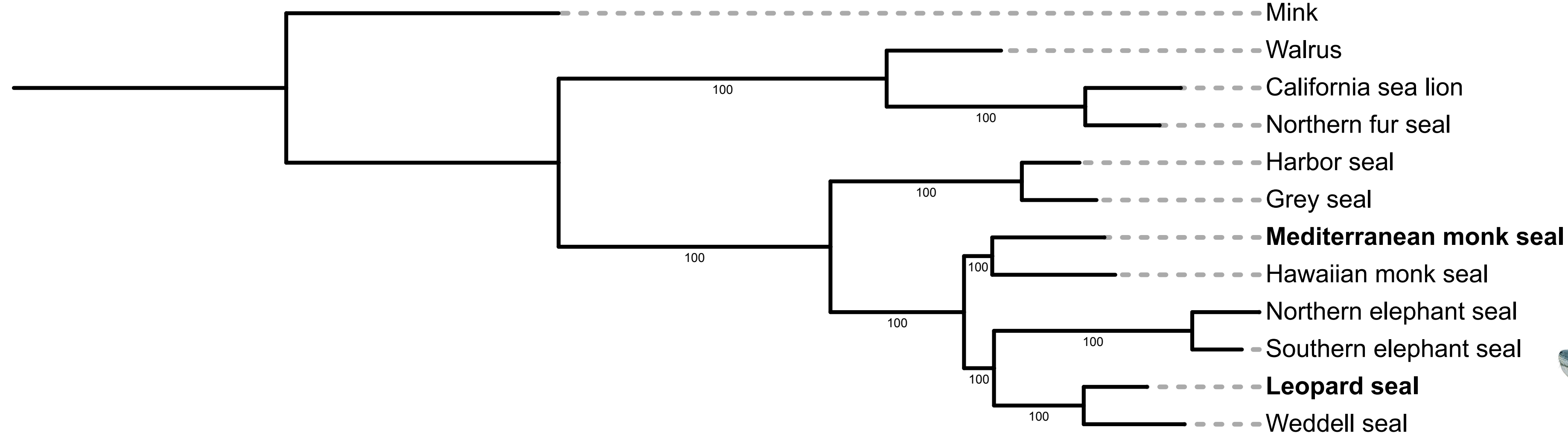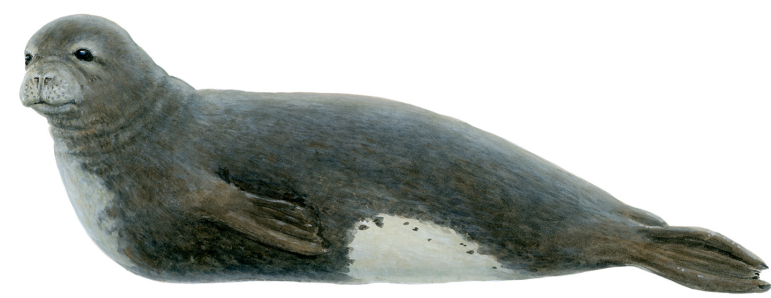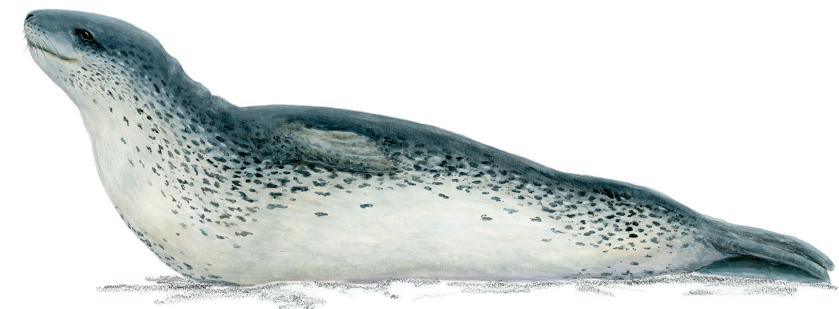

Supplement: Supplementary file 4 — Additional file 4: Fig. S4 Maximum likelihood phylogeny. [file 12915_2025_2207_MOESM4_ESM.pdf]
