# Supplementary material for: Mediterranean monk seal (Monachus monachus) and leopard seal (Hydrurga leptonyx) de novo genomes to study the demographic history and genetic diversity of southern seals
Source: BMC Biol. 2025 Apr 16;23:102. doi: 10.1186/s12915-025-02207-w (PMC12004778; doi:10.1186/s12915-025-02207-w)
Supplement: Supplementary file 5 — Additional file 5: Table S5 Mediterranean monk seal sample overview. [file 12915_2025_2207_MOESM5_ESM.docx]

**Supplementary table 5 Mediterranean monk seal sample overview**

| **Sample name** | **Tissue** | **Sex** |
| --- | --- | --- |
| M-2 | Skin and blubber | Male |
| M-4 | Skin and blubber | Female |
| M12 | Skin and blubber | Female |
| M-13 | Skin and blubber | Female |
| M-15 | Skin and blubber | Male |
