# Supplementary material for: Mediterranean monk seal (Monachus monachus) and leopard seal (Hydrurga leptonyx) de novo genomes to study the demographic history and genetic diversity of southern seals
Source: BMC Biol. 2025 Apr 16;23:102. doi: 10.1186/s12915-025-02207-w (PMC12004778; doi:10.1186/s12915-025-02207-w)
Supplement: Supplementary file 6 — Additional file 6: Table S6 Publication record monk seal samples. [file 12915_2025_2207_MOESM6_ESM.docx]

**Supplementary Table 6 Summary of all scientific publications the Mediterranean monk seal samples of the underlying study have been part of.**

1.      Borrell, A.; Aguilar, A. & Pastor, T. (1997): Organochlorine pollutant levels in Mediterranean monk seals from the western Mediterranean and the Sahara coast. Marine Pollution Bulletin 34(7):505-510.

2.      Hernández, M.; Robinson, I.; Aguilar, A.; González, L. M.; López-Jurado, L. F.; Reyero, M. I.; Cacho, E.; Franco, J.; López-Rodas, V.; Costas, E. (1998): Did algal toxins cause monk seal mortality? Nature (London), 393.28-29.

3.      Pastor, T.; Garza, J. C.; Allen, P.; Amos, W. & Aguilar, A. (2004): Low genetic variability in the highly endangered Mediterranean monk seal. Journal of Heredity, 95(4): 291-300.

4.      Fyler, C. A.; Reeder, T. W.; Berta, A.; Antonelis, G.; Aguilar, A. & Androukaki, E. (2005): Historical biogeography and phylogeny of monachine seals (Pinnipedia: Phocidae) based on mitochondrial and nuclear DNA data. Journal of Biogeography, 32:1267-1279.

5.      Borrell, A., Cantos, G., Aguilar, A., Androukaki, E. and Dendrinos, P. (2007) Concentrations and patterns of organochlorine pesticides and PCBs in Mediterranean monk seals (Monachus monachus) from Western Sahara and Greece. Science of the Total Environment, 381:316-325.

6.      Pastor, T.; Garza, J. C., Aguilar, A., Tounta, E. & Androukaki, E. (2007): Genetic diversity and differentiation between the two remaining populations of the critically endangered Mediterranean monk seal. Animal Conservation, 10: 461-469.

7.      Karamanlidis, A. A.; Gaughran, S.; Aguilar, A.; Dendrinos, P.; Huber, D.; Pires, R.;  Schultz, J.; Skrbinšekh, T. & Amato, G. (2016): Shaping species conservation strategies using mtDNA analysis: the case of the elusive Mediterranean monk seal (Monachus monachus). Biological Conservation, 193: 71-79.

8.      Gaubert, P.; Justy, F.; Mo, G.; Aguilar, A.; Danyer, E.; Borrell, A.; Dendrinos, P.; Öztürk, B.; Improta, R.; Tonay, A. M.; Karamanlidis, A. A. (2019): Insights from 180 years of mitochondrial variability in the endangered Mediterranean monk seal (Monachus monachus). Marine Mammal Science, 35(4): 1489-1511. DOI: 10.1111/mms.12604.

9.      Dayon, J., Lecompte, E.; Aguilar, A.; Fernandez de Larrinoa, P.; Pires, R.; Gaubert, P. (2020) Development and characterization of nineteen microsatellite loci for the endangered Mediterranean monk seal Monachus monachus. Marine Biodiversity, 50(5): 1-16.

10.     Rey-Iglesia, A.; Gaubert, P.; Espregueira-Themudo, G.; Pires, R.; de la Fuente, C.; Freitas, L.; Aguilar, A.; Borrell, A.; Krakhmalnaya, T.; Vasconcelos, R.; Campos, P. F. (2021): Mitogenomics of the endangered Mediterranean monk seal (Monachus monachus) reveals dramatic loss of diversity and supports historical gene flow between Atlantic and eastern Mediterranean populations. Zoological Journal of the Linnean Society ,191(4), 1147-1159.

11.     Salmona, J.; Dayon, J.; Lecompte, E.; Karamanlidis, A. A.; Aguilar, A.; Fernandez De Larrinoa, P.; Pires, R.; Mo, G.; Panou, A.; Agnesi, S.; Borrell, A.; Danyer, E.; Öztürk, B.; Tonay, A. M.; Anestis, A. K.; González, L. M.; Dendrinos, P.; Gaubert, P. (2022): The antique genetic plight of the Mediterranean monk seal (Monachus monachus). Proceedings of the Royal Society B, 289: 20220846.
