# Supplementary material for: Mediterranean monk seal (Monachus monachus) and leopard seal (Hydrurga leptonyx) de novo genomes to study the demographic history and genetic diversity of southern seals
Source: BMC Biol. 2025 Apr 16;23:102. doi: 10.1186/s12915-025-02207-w (PMC12004778; doi:10.1186/s12915-025-02207-w)
Supplement: Supplementary file 7 — Additional file 7: Table S7 Seal genomes and genome data taken from databases. [file 12915_2025_2207_MOESM7_ESM.docx]

**Supplementary table 7 Available data from databases taken for this study (NCBI Accession, BioProject number, year of submission & linked publications)**

| **Species** | **Accession Number / Bioproject** | **Publications** |
| --- | --- | --- |
| **European mink**  (*Mustela lutreola*) | GCF_030435805.1  PRJNA984926 (2023)  https://www.ncbi.nlm.nih.gov/bioproject/PRJNA984926/ | Skorupski J *et al.,* "Prioritizing Endangered Species in Genome Sequencing: Conservation Genomics in Action with the First Platinum-Standard Reference-Quality Genome of the Critically Endangered European Mink Mustela lutreola L., 1761.", *Int J Mol Sci*, 2023 Oct 1;24(19)  Rhie A *et al.,* "Towards complete and error-free genome assemblies of all vertebrate species.", *Nature*, 2021 Apr;592(7856):737-746 |
| **Walrus**  (*Odobenus rosmarus*) | GCF_000321225.1  PRJNA167474 (2012)  https://www.ncbi.nlm.nih.gov/bioproject/PRJNA167474/ | Foote AD *et al.,* "Convergent evolution of the genomes of marine mammals.", *Nat Genet*, 2015 Mar;47(3):272-5  Arnason U *et al.,* "Mammalian mitogenomic relationships and the root of the eutherian tree.", *Proc Natl Acad Sci U S A*, 2002 Jun 11;99(12):8151-6 |
| **Northern elephant seal**  (*Mirounga angustirostris*) | GCF_021288785.2, SRX10915207  PRJNA512907 (2019) | Moreno J *et al.,* "Emx2 underlies the development and evolution of marsupial gliding membranes", *Nature*, 2024;629(8010):127-135 |
| **Southern elephant seal**  (Mirounga leonina) | GCF_011800145.1  PRJNA587380 (2020) | Pinniped phylogeny and a new hypothesis for their origin and dispersal.", *Mol Phylogenet Evol*, 2006 Nov;41(2):345-54 |
| **Hawaiian monk seal**  (*Neomonachus schauinslandi*) | GCF_002201575.2, SRX20210922  PRJNA384558 (2017) | - |
| **Weddell seal**  (*Leptonychotes weddellii*) | GCF_000349705.1  PRJNA68235 (2013) | Pinniped phylogeny and a new hypothesis for their origin and dispersal.", *Mol Phylogenet Evol*, 2006 Nov;41(2):345-54 |
| **Grey seal**  (*Halichoerus grypus*) | GCF_012393455.1, SRX12373285  PRJNA577240 (2020) | - |
| **Harbor seal**  (*Phoca vitulina*) | GCF_004348235.1  PRJNA494181 (2019) | Nilsson MA *et al.,* "Radiation of extant marsupials after the K/T boundary: evidence from complete mitochondrial genomes.", *J Mol Evol*, 2003;57 Suppl 1:S3-12  Arnason U *et al.,* "The complete mitochondrial DNA sequence of the harbor seal, Phoca vitulina.", *J Mol Evol*, 1992 Jun;34(6):493-505 |
| **California sea lion**  (*Zalophus californianus*) | GCA_004024565.1  PRJNA399369 (2019) | - |
| **Northern fur seal**  (*Callorhinus ursinus*) | GCF_003265705.1  PRJNA475116 (2018) | - |
| **Antarctic fur seal**  (*Arctocephalus gazella*) | GCA_900642305.1  PRJEB30577 (2019) | - |
| **Steller sea lion**  (*Eumetopias jubatus*) | GCF_004028035.1  PRJNA475770 (2019) | Kwan HH *et al.,* "The Genome of the Steller Sea Lion (Eumetopias jubatus).", *Genes (Basel)*, 2019 Jun 26;10(7)  Arnason U *et al.,* "Mammalian mitogenomic relationships and the root of the eutherian tree.", *Proc Natl Acad Sci U S A*, 2002 Jun 11;99(12):8151-6 |
| **Crabeater seal**  (Lobodon carcinophaga) | SRX2987924  PRJNA389902 (2017) | Leonardi, et al. Phylogenomic analysis of seal lice reveals codivergence with their hosts. Systematic Entomology 44: 4. |
