# Supplementary material for: Mediterranean monk seal (Monachus monachus) and leopard seal (Hydrurga leptonyx) de novo genomes to study the demographic history and genetic diversity of southern seals
Source: BMC Biol. 2025 Apr 16;23:102. doi: 10.1186/s12915-025-02207-w (PMC12004778; doi:10.1186/s12915-025-02207-w)
Supplement: Supplementary file 8 — Additional file 8: Table S8 Mapping quality of reference-based assemblies. [file 12915_2025_2207_MOESM8_ESM.docx]

**Supplementary Table 8 Mapping statistics of all reference-based assemblies**

Mapping statistics of the reference-based assemblies of the newly sequenced leopard seal and Mediterranean monk seals, together with the short-read data taken from databases estimated with Qualimap. All reads were mapped against the *de novo* genome of the Mediterranean monk seal.

| **Sample** | **Mapped reads**  **[no / %]** | **Av. mapping quality** | **Error rate** | **Av. coverage** |
| --- | --- | --- | --- | --- |
| M. monk seal-2 | 252,055,380 (100%) | 55.8 | 0.0024 | 15.6 |
| M. monk seal-4 | 198,962,828 (100%) | 55.2 | 0.0074 | 11.9 |
| M. monk seal-12 | 182,221,295 (100%) | 55.0 | 0.0020 | 11.0 |
| M. monk seal-13 | 485,553,891 (99.5%) | 54.8 | 0.0020 | 16.3 |
| M. monk seal-15 | 200,488,282 (100%) | 55.9 | 0.0020 | 12.0 |
| Leopard seal | 486,047,686 (99.7%) | 47.1 | 0.0044 | 30.5 |
| N. elephant seal | 95,263,640 (100%) | 52.7 | 0.0192 | 9.1 |
| H. monk seal | 95,437,174 (100%) | 53.0 | 0.0150 | 5.9 |
| Weddell seal | 307,356,502 (100%) | 52.6 | 0.0160 | 19.4 |
| Crabeater seal | 114,938,185 (100%) | 52.9 | 0.0157 | 7.2 |
