# Supplementary material for: Mediterranean monk seal (Monachus monachus) and leopard seal (Hydrurga leptonyx) de novo genomes to study the demographic history and genetic diversity of southern seals
Source: BMC Biol. 2025 Apr 16;23:102. doi: 10.1186/s12915-025-02207-w (PMC12004778; doi:10.1186/s12915-025-02207-w)
Supplement: Supplementary file 9 — Additional file 9: Table S9 Calibration points used for dating the phylogeny. [file 12915_2025_2207_MOESM9_ESM.docx]

**Supplementary Table 9 Calibration points used for generating the time-calibrated phylogeny with MCMCtree.** Calibration reading was taken over from supplementary material of Fulton & Strobeck (2009). Studies about the fossil origins are linked in the table.

| **Calibration** | **Median age [Ma]** | **95% range [Ma]** | **Source** |
| --- | --- | --- | --- |
| Musteloidea-  Pinnipedia | 29.95 | 26.57 - 40.66 | Berta, 1991; Wang et al., 2005; Finarelli, 2008; Baskin, 1998 |
| Otariidae-  Odobenidae | 17.56 | 14.91 - 23.69 | Kohno et al., 1994; Demere et al., 2003 |
| *Halichoerus*-  *Phoca*-*Pusa* | 4.982 | 1.365 - 23.72 | Demere et al., 2003 |
| Lobodontini-  Miroungini | 9.959 | 6.826 - 15.75 | Muizon, 1982; de Muizon & DeVries, 1985; Demere et al., 2003 |
